# Occupational exposure risk perception and its multilevel influencing factors among nurses in central sterile supply departments: a cross-sectional study based on the social ecological model

**DOI:** 10.3389/fpubh.2026.1786625

**Published:** 2026-04-01

**Authors:** Yu Luo, Xin Huang, Bangyan Guan, Jiarong Tan, Feng Gao

**Affiliations:** 1 Department of Nursing, Chongqing Public Health Medical Center, Chongqing, China; 2 Department of Digitalization, Chong Gang General Hospital, Chongqing, China; 3 Cancer Center, Chongqing University Three Gorges Hospital, Chongqing, China

**Keywords:** central sterile supply department, influencing factors, occupational exposure, risk perception, social ecological model

## Abstract

**Objective:**

Occupational exposure remains a critical issue in occupational safety management within healthcare institutions. Nurses working in central sterile supply departments (CSSDs) face a relatively high risk of occupational exposure due to the unique characteristics of their working environment. However, the multilevel mechanisms influencing occupational exposure risk perception among this population have not been systematically investigated. This study aimed to explore the level of occupational exposure risk perception and its multilevel influencing factors among CSSD nurses.

**Methods:**

A cross-sectional study design was employed. From June 2025 to January 2026, study preparation and site coordination were conducted. Following ethics approval in January 2026, an online questionnaire survey was administered among 580 CSSD nurses from 28 tertiary hospitals. Guided by the Social Ecological Model, variables were collected across individual, interpersonal, organizational, community, and policy levels. Stepwise multiple linear regression analysis was performed to identify factors associated with occupational exposure risk perception.

**Results:**

The overall level of occupational exposure risk perception among CSSD nurses was moderate, tending to be above the midpoint of the scale. Multivariate analysis showed that self-efficacy, social support, participation in occupational exposure training, regular emergency drills, and supervisory protection were positively associated with occupational exposure risk perception, whereas occupational burnout was negatively associated with risk perception (all *p* < 0.05).

**Conclusion:**

Occupational exposure risk perception among CSSD nurses was associated with individual psychological resources and organizational support factors. Based on these findings, multilevel strategies may be considered and should be evaluated in future longitudinal or interventional studies, including strengthening psychological resource support, occupational safety training, and organizational safety culture, to inform capacity building in occupational protection and infection prevention and control.

## 1 Introduction

The central sterile supply department (CSSD) is a critical hub within hospital infection prevention and control systems, responsible for the cleaning, disinfection, sterilization, and distribution of medical instruments. The quality of CSSD operations directly affects patient safety and the overall quality of healthcare services (1). CSSD nurses work long-term in complex environments characterized by high temperature, high humidity, chemical disinfectants, and potential biological contamination, making them particularly vulnerable to occupational hazards such as sharps injuries, chemical injuries, and infectious exposures. Consequently, CSSD nurses may face substantial occupational exposure risks due to the unique characteristics of their working environment (2). Previous studies have suggested that occupational exposure is associated with psychological distress, personal protective equipment compliance, and work-related outcomes among healthcare workers. Those with a higher occupational exposure burden tend to report higher levels of psychological stress, which may be accompanied by poor compliance with protective measures and negative work performance outcomes (3). Risk perception (RP), defined as an individual’s subjective assessment of the likelihood of potential hazards and the severity of their consequences, is a key psychological determinant of occupational protective behavior (4). Studies have shown that a higher level of risk perception is associated with more proactive safety and protective behaviors, whereas lower risk perception is often related to insufficient protective behaviors or inadequate implementation of protective measures (5).

However, existing studies on occupational exposure risk perception have predominantly focused on nurses working in high-risk clinical settings such as emergency departments and infectious disease units. Research specifically targeting CSSD nurses remains limited, and their levels of occupational exposure risk perception as well as the associated influencing factors have yet to be clearly elucidated (6). Occupational exposure risk perception is shaped by multiple determinants, encompassing individual characteristics such as age, years of work experience, educational level, and self-efficacy, as well as external factors including organizational safety culture, management systems, and occupational training (7, 8).

The Social Ecological Model (SEM) emphasizes that health-related cognitions and behaviors are not determined by a single factor, but rather are jointly shaped through interactions among multiple levels, including the individual, interpersonal, organizational, community, and policy environments (9). Occupational exposure risk perception among CSSD nurses also shows a clear multilevel pattern. At the individual level, risk perception may be influenced by psychological and social resources such as self-efficacy, occupational burnout, and social support. At the interpersonal level, reminders from colleagues and guidance or supervision from supervisors may affect the implementation of protective practices. At the organizational level, safety management measures—including occupational exposure training, provision of emergency supplies, regular drills, and the convenience of reporting procedures—may influence nurses’ ability to identify and respond to risks. At broader community and policy levels, professional initiatives, experience sharing, as well as institutional standards and reward–penalty mechanisms may also shape individuals’ judgments about occupational exposure risks and their selection of protective behaviors.

Accordingly, this study adopted SEM as the theoretical framework, treated occupational exposure risk perception among CSSD nurses as the outcome, and systematically integrated candidate determinants across the five levels for operational measurement and statistical analysis. This approach aimed to elucidate multilevel correlates and their association patterns, thereby informing the development and rigorous evaluation of coordinated multilevel strategies targeting individual psychological resources, organizational support, and institutional governance.

## 2 Methods

### 2.1 Study design and participants

From June 2025 to January 2026, study preparation and site engagement were completed. After ethics approval in January 2026, an online cross-sectional survey was conducted among in-service CSSD nurses from 28 tertiary hospitals in Chongqing, China. Participating hospitals were identified through feasibility-based convenience recruitment. Specifically, tertiary hospitals in Chongqing that had the capacity to implement the study and agreed to participate were invited, and a total of 28 tertiary hospitals were ultimately included. Within each participating hospital, the CSSD served as the cluster unit, and cluster invitation within hospital was used to invite all on-duty CSSD nurses to complete the questionnaire. The head of the CSSD or the nurse manager in each hospital acted only as a liaison, responsible for forwarding a standardized study information sheet and the survey link, without any individual-level screening or allocation. Participation was voluntary and anonymous, and respondents could withdraw at any time. The inclusion criteria were as follows: (1) having worked in the CSSD for at least 6 months; (2) holding a valid Nurse Practice Certificate and possessing independent operational competence; (3) being informed of the study objectives and providing voluntary participation; (4) having basic reading ability and the capacity to understand the questionnaire. The exclusion criteria were: (1) interns, trainees, or non-nursing personnel; (2) individuals on long-term leave or transferred from their original positions; (3) individuals with evident cognitive impairment or diagnosed mental disorders. According to the sample size estimation principle for multivariate linear regression analysis, the required sample size should be 10–20 times the number of independent variables. In this study, a total of 27 independent variables—including demographic characteristics, psychological factors, and organizational management–related variables—were included, resulting in a theoretically required sample size of 270–540 participants. Considering potential invalid questionnaires and the need for stratified analyses, the sample size was further expanded. Ultimately, 580 CSSD nurses were surveyed, and all participants provided online informed consent prior to participation. This study was approved by the Ethics Committee of Chongqing Public Health Medical Center (Approval No. 2026-002-01-KY).

### 2.2 Research instruments

Guided by the Social Ecological Model (SEM), this study measured variables at five levels (individual, interpersonal, organizational, community, and policy) to assess the level of occupational exposure risk perception among nurses working in CSSDs and to identify its influencing factors.

The measurement tools in this study comprised two components. First, a set of standardized scales was used, including the Occupational Exposure Risk Perception Scale for CSSD nurses (as the outcome), the General Self-Efficacy Scale (GSES), the Chinese Maslach Burnout Inventory (CMBI), and the Social Support Rating Scale (SSRS), to assess individual-level risk perception as well as psychological and social resource indicators. Based on the five levels of the SEM, this study developed a context-specific item module to assess factors at the interpersonal, organizational, community, and policy levels. The items were drafted by the research team with reference to key steps in the prevention and control process of occupational exposure in CSSDs and relevant literature, and were finalized after review by clinical experts for wording and content relevance. The module was designed to improve the contextual fit of the measures to the occupational exposure experiences of CSSD nurses. Given the multicenter nature of the survey and the need to balance feasibility with respondent burden, most items in this module were measured using dichotomous response options (yes/no).

#### 2.2.1 Individual level

(1) General information and occupational exposure experience.

Information was collected on nurses’ sex, age, educational level, professional title, years of work in CSSD, shift pattern, and night-shift frequency. Occupational exposure events during the past year were recorded, including sharps injuries, chemical exposure, scald/steam burns, and body fluid splashes. In addition, we assessed post-exposure management and individual protection-related knowledge and competencies, including whether reporting and initial management were completed, whether follow-up was completed, whether appropriate protective equipment could be correctly selected, whether emergency response capability was in place, and whether nurses were familiar with chemical safety exposure limits.

(2) Occupational Exposure Risk Perception Scale for CSSD Nurses

Occupational exposure risk perception was measured using a scale developed by Zhu et al. (10) based on risk perception theory and health behavior change theory, specifically designed for CSSD nurses. The scale comprises 33 items across five dimensions: perceived likelihood of occupational exposure (items 1–7), perceived severity of exposure consequences (items 8–14), participatory dialogue (items 15–20), behavioral confidence (items 21–27), and environmental change (items 28–33). Items are rated on a 5-point Likert scale ranging from 1 (strongly disagree) to 5 (strongly agree). Total scores range from 33 to 165, with higher scores indicating higher levels of occupational exposure risk perception. The scale demonstrated good reliability, with a Cronbach’s *α* of 0.869 and a test–retest reliability of 0.919. As no widely accepted cutoffs or normative criteria have been established in the scale development and validation literature for categorizing scores into low, moderate, or high levels, an operational classification based on the mean item score was applied in the present study using equal-interval partitioning: low (1.00–2.33), moderate (2.34–3.66), and high (3.67–5.00). The mean item score preserves the original 5-point response metric and facilitates intuitive interpretation of the overall level. This classification was used for descriptive purposes only and was not applied as a diagnostic threshold or in inferential analyses.

(3) General Self-Efficacy Scale (GSES)

General self-efficacy was assessed using the General Self-Efficacy Scale (GSES), originally developed by Schwarzer et al. (11) and later revised and validated in China by Wang et al. (12). The scale consists of 10 items assessing individuals’ confidence and perceived control when facing difficulties or challenges. Items are rated on a 4-point Likert scale ranging from 1 (not at all true) to 4 (exactly true). The total score ranges from 10 to 40, with higher scores indicating stronger self-efficacy. In this study, the Cronbach’s *α* coefficient of the scale was 0.931 (12).

(4) Chinese Maslach Burnout Inventory (CMBI)

Occupational burnout was measured using the Chinese Maslach Burnout Inventory (CMBI), revised by Li et al. (13) based on the Maslach Burnout Inventory (MBI) and widely applied in studies of occupational mental health among Chinese healthcare workers. The CMBI contains 15 items across three dimensions: emotional exhaustion (items 1–5), depersonalization (items 6–10), and reduced personal accomplishment (items 11–15). The “reduced personal accomplishment” dimension was reverse-scored. Items are rated on a 7-point Likert scale ranging from 1 (never) to 7 (every day). The total score ranges from 15 to 105, and higher scores across each dimension indicate more severe occupational burnout. The Cronbach’s *α* coefficients reported for the scale among healthcare workers range from 0.80 to 0.90 (14).

(5) Social Support Rating Scale (SSRS)

Social support was assessed using the Social Support Rating Scale (SSRS) developed by Xiao (15). The scale includes 10 items covering three dimensions: subjective support (items 2, 6, and 7), objective support (items 1, 3, 4, and 5), and utilization of support (items 8, 9, and 10). The total score ranges from 10 to 66, with higher scores indicating better social support. The SSRS has been widely used in different populations in China, with reported Cronbach’s *α* coefficients ranging from 0.89 to 0.94 (16).

The GSES, CMBI, and SSRS were operationalized as individual-level psychological and social resource indicators and were entered into the analyses using their raw total scores as continuous variables. All scores were calculated strictly in accordance with the original scoring manuals. Specifically, the occupational exposure risk perception scale total score was obtained by summing item scores, and subscale scores were also calculated for descriptive reporting. The GSES total score was computed as the sum of item scores. For the CMBI, scores were calculated for the three subscales (emotional exhaustion, depersonalization, and reduced personal accomplishment); the reduced personal accomplishment subscale was reverse-coded as required, and an overall CMBI total score was additionally derived for descriptive reporting and modeling. For the SSRS, the total score and three subscale scores (subjective support, objective support, and support utilization) were calculated according to the scale guidelines. All scale scores were summarized descriptively as continuous variables (see “Section 3.2”) and were further included in correlation analyses and multivariable linear regression models in line with the study objectives.

#### 2.2.2 Interpersonal level

The interpersonal level assessed peer and supervisory support in the workplace using two items: “whether colleagues remind staff to comply with protective procedures” and “whether supervisors monitor protective practices.” Responses were rated on a dichotomous (yes/no) scale and coded as yes = 1 and no = 0 for subsequent statistical analyses.

#### 2.2.3 Organizational level

The organizational level primarily assessed the extent of protective support available to nurses from the perspective of managerial practices and institutional arrangements. Five items were included: “whether they had received occupational exposure training,”“whether the department was equipped with emergency protective supplies,”“whether protective supplies were sufficient,”“whether regular occupational exposure drills were conducted,” and “whether the reporting procedure was convenient.” Responses were recorded dichotomously (yes/no) and coded as yes = 1 and no = 0 for subsequent statistical analyses.

#### 2.2.4 Community level

The community level reflected support from professional organizations and external industry groups. Two items were included: “whether advocacy from nursing associations increases attention to protection” and “whether peer experience sharing enhances protective awareness.” Responses were recorded dichotomously (yes/no) and coded as yes = 1 and no = 0 for subsequent statistical analyses.

#### 2.2.5 Policy level

The policy level reflected macro-level institutional and regulatory factors. Two items were included: “whether nurses are familiar with hospital and national occupational exposure management standards” and “whether the hospital has reward and punishment mechanisms for the implementation of protective measures.” Responses were recorded dichotomously (yes/no) and coded as yes = 1 and no = 0 for subsequent statistical analyses.

#### 2.2.6 SEM-based classification and coding of candidate independent variables

In this study, candidate independent variables were categorized according to the five-level SEM framework. The individual level comprised general characteristics, occupational exposure experiences, post-exposure management, items assessing personal protective knowledge/competencies, and scale-based measures including GSES, CMBI, and SSRS. The interpersonal, organizational, community, and policy levels corresponded to the items described above. Scale scores were entered into the regression analyses as continuous variables using total scores. Dichotomous items were coded as yes/no (yes = 1, no = 0). For categorical variables in the general characteristics section, a reference category was specified when incorporating them into the models. Variable names and their SEM-level assignments in the results tables were consistent with those described in this section.

### 2.3 Data collection

This study was conducted as an online anonymous survey via the Wenjuanxing platform. After ethics approval was obtained in January 2026, the research team first contacted the heads of the CSSDs in the 28 participating hospitals and obtained departmental approval. Subsequently, the designated CSSD liaison at each hospital, typically a head nurse or department head, disseminated a standardized study information sheet and the questionnaire link to all CSSD nurses on duty through departmental work groups or internal notices. The information sheet stated that participation was entirely voluntary and anonymous, would not affect performance appraisal or evaluation, and that participants could withdraw at any time without any incentives or penalties. To ensure data quality, mandatory items and logic checks were embedded in the questionnaire, and clearly invalid or duplicate responses were removed. In total, 600 questionnaires were distributed and 580 valid questionnaires were returned, yielding an effective response rate of 96.67%.

### 2.4 Statistical analysis

Statistical analyses were performed using SPSS version 26.0(IBM Corp, Armonk, NY, USA). Prior to data entry, two researchers independently checked the data to ensure accuracy. Continuous variables were tested for normality and are presented as mean±standard deviation (SD), while categorical variables are presented as frequencies and percentages (*n*, %). Independent-samples t tests or one-way analysis of variance (ANOVA) were used to compare differences in occupational exposure risk perception scores across groups with different general characteristics. The total occupational exposure risk perception score was used as the dependent variable. Candidate predictors at the individual level (e.g., sociodemographic and work-related characteristics, occupational exposure experiences, and scores reflecting individual psychological and social resources), together with variables from other SEM levels, were included in the analytical framework. Variables showing statistical significance in the univariate analyses (*p* < 0.05) were entered into a stepwise multiple linear regression model, with occupational exposure risk perception score as the dependent variable. The criteria for variable entry and removal were set at *p* < 0.05 and *p* > 0.10, respectively. Collinearity diagnostics were performed, and variance inflation factor (VIF) values<5 were considered to indicate no obvious multicollinearity. All statistical tests were two-tailed, and a *p* value < 0.05 was considered statistically significant.

## 3 Results

### 3.1 Common method bias test

Common method bias was assessed using Harman’s single-factor test. The results showed that the largest common factor accounted for 42.62% of the total variance, which did not exceed the 50% threshold (17), suggesting that the risk of common method bias in this study was likely low.

### 3.2 Individual-level scale scores (occupational exposure risk perception, occupational burnout, self-efficacy, and social support)

The individual-level scale scores among nurses working in CSSDs are presented in Table 1. Beyond individual-level psychological and social resources, items at the interpersonal, organizational, community, and policy levels were also included based on the SEM framework. Their associations with risk perception were further examined in both univariate analyses and the multivariable linear regression model (see Tables 2, 3).

**Table 1:** Scores of occupational exposure risk perception, occupational burnout, self-efficacy, and social support among CSSD nurses (*n* = 580, mean±SD).

Item	Total score (mean ± SD)
Overall risk perception	113.14 ± 17.12
Likelihood of occupational exposure	19.11 ± 6.83
Severity of occupational exposure consequences	23.50 ± 6.36
Participatory dialogue	18.16 ± 4.80
Behavioral confidence	30.60 ± 4.15
Environmental change	21.80 ± 4.15
Self-efficacy	30.54 ± 4.04
Overall occupational burnout	53.52 ± 10.58
Emotional exhaustion	17.39 ± 4.49
Depersonalization	15.84 ± 4.26
Reduced personal accomplishment	20.29 ± 5.91
Overall social support	21.13 ± 4.06
Subjective support	8.71 ± 1.94
Objective support	8.19 ± 2.40
Utilization of support	4.23 ± 1.49

**Table 2:** Univariate analysis of occupational exposure risk perception among CSSD nurses (*n* = 580, mean±SD).

Variable	*n*	Score (mean ± SD)	t/*F* value	*p* value
Age			6.902	0.001*
<30 years	168	109.72 ± 16.28		
30–40 years	238	113.88 ± 15.73		
>40 years	174	115.43 ± 18.03		
Gender			0.785	0.433
Male	93	114.05 ± 17.21		
Female	487	112.47 ± 17.12		
Education			0.367	0.693
College	228	111.52 ± 16.33		
Bachelor	342	114.62 ± 17.52		
Graduate or above	10	117.10 ± 18.48		
Professional title			0.921	0.431
Nurse	130	109.52 ± 15.92		
Nurse practitioner	198	112.97 ± 16.74		
Supervisor nurse	180	115.86 ± 17.63		
Deputy chief nurse or above	72	118.44 ± 19.21		
Years of work in CSSD			9.23	<0.001*
<5 years	189	109.64 ± 16.25		
5–10 years	205	113.23 ± 15.83		
>10 years	186	117.06 ± 18.10		
Shift pattern			−1.70	0.089
Fixed day shift	140	111.38 ± 17.63		
Rotating shifts	440	114.26 ± 17.09		
Night shift frequency			8.670	<0.001*
0 times/month	140	109.47 ± 15.54		
1–4 times/month	312	111.95 ± 16.82		
≥5 times/month	128	117.15 ± 18.12		
Received occupational exposure training			−5.113	<0.001*
No	159	107.31 ± 15.98		
Yes	421	115.34 ± 17.12		
Emergency protective equipment available in the department			4.807	<0.001*
No	64	104.21 ± 15.81		
Yes	516	114.37 ± 17.03		
Sharps injury			−2.097	0.037*
No	418	113.75 ± 17.48		
Yes	162	110.52 ± 16.31		
Chemical exposure			−2.907	0.004*
No	461	113.91 ± 17.36		
Yes	119	109.13 ± 15.62		
Scald/steam injury			−1.44	0.151
No	420	113.68 ± 17.44		
Yes	160	111.42 ± 16.83		
Body fluid splash			−3.47	0.001*
No	464	114.59 ± 17.18		
Yes	116	108.76 ± 16.97		
Completed reporting and management after exposure			−8.362	<0.001*
No	189	104.37 ± 15.63		
Yes	391	117.38 ± 16.92		
Post-exposure follow-up			4.363	<0.001*
No	191	108.16 ± 16.47		
Yes	389	114.74 ± 17.05		
Correct selection of protective equipment			5.096	<0.001*
No	75	104.86 ± 15.33		
Yes	505	114.68 ± 17.11		
Emergency response capability			3.564	0.001*
No	93	107.48 ± 16.01		
Yes	487	114.01 ± 17.03		
Familiarity with chemical safety limits			3.060	0.002*
No	91	108.53 ± 15.89		
Yes	489	114.51 ± 17.29		
Colleagues’ reminders for protection			3.042	0.003*
No	52	107.36 ± 15.41		
Yes	528	114.24 ± 17.04		
Supervisory oversight of protection			4.846	<0.001*
No	73	106.21 ± 16.03		
Yes	507	114.14 ± 17.17		
Adequate protective supplies			3.366	0.001*
No	55	106.54 ± 16.32		
Yes	525	114.36 ± 17.06		
Regular occupational exposure drills			−4.645	<0.001*
No	88	106.12 ± 15.95		
Yes	492	114.39 ± 17.10		
Streamlined reporting procedure			4.24	<0.001*
No	107	107.13 ± 16.20		
Yes	473	114.61 ± 17.08		
Influence of nursing association advocacy			2.16	0.031*
No	110	110.08 ± 16.69		
Yes	470	113.82 ± 17.01		
Peer experience sharing			−4.565	<0.001*
No	232	109.17 ± 16.33		
Yes	348	115.79 ± 17.17		
Familiarity with the occupational exposure management protocol			5.01	<0.001*
No	97	106.31 ± 15.77		
Yes	483	115.06 ± 17.09		
Clear reward and punishment mechanisms			4.85	<0.001*
No	113	107.48 ± 15.78		
Yes	467	115.27 ± 17.23		

Independent-samples *t*-tests were used for mean comparisons between two groups, and ANOVA was applied for comparisons among three or more groups. The “t/F” statistic is reported as a t value or an F value depending on the test used.

*Indicates *p* < 0.05.

**Table 3:** Stepwise multiple linear regression analysis of factors associated with occupational exposure risk perception.

Independent variable	B	SE	*β*	*t*	*p* value	VIF
Constant	58.213	4.275	—	13.626	<0.001	—
Self-efficacy (GSES total score)	0.417	0.098	0.291	4.264	<0.001	1.372
Occupational burnout (CMBI total score)	−0.325	0.107	−0.265	−3.046	0.003	1.456
Social support (SSRS total score)	0.289	0.092	0.236	3.141	0.002	1.328
Received occupational exposure training (yes vs. no [Ref])	4.813	1.571	0.182	3.063	0.002	1.217
Regular occupational exposure drills (yes vs. no [Ref])	4.107	1.536	0.163	2.676	0.008	1.304
Supervisory oversight of protection(yes vs. no [Ref])	3.267	1.521	0.147	2.148	0.034	1.246

Model fit was *R*^2^ = 0.532 and adjusted *R*^2^ = 0.527 (*F* = 108.560, *p* < 0.001). B denotes the unstandardized regression coefficient, SE the standard error, and β the standardized regression coefficient. For dichotomous variables, “no” served as the reference category (Ref). VIFs were examined to assess multicollinearity.

### 3.3 Univariate analysis of occupational exposure risk perception among CSSD nurses

The results of the univariate analysis of occupational exposure risk perception among nurses working in Central Sterile Supply Departments (CSSDs) are presented in Table 2.

### 3.4 Correlation analysis between occupational exposure risk perception and self-efficacy, occupational burnout, and social support among CSSD nurses

The correlations between occupational exposure risk perception and self-efficacy, occupational burnout, and social support among nurses working in CSSDs are presented in Table 4.

**Table 4:** Correlation analysis between occupational exposure risk perception and self-efficacy, occupational burnout, and social support among CSSD nurses.

Variable	Self-efficacy	Occupational burnout	Emotional exhaustion	Depersonalization	Reduced personal accomplishment	Social support	Subjective support	Objective support	Utilization of support
Overall risk perception	0.326**	−0.284**	−0.297**	−0.241**	−0.218**	0.301**	0.278**	0.266**	0.245**
Likelihood of occupational exposure	0.294**	−0.231**	−0.256**	−0.210**	−0.196**	0.275**	0.252**	0.230**	0.213**
Severity of occupational exposure consequences	0.303**	−0.297**	−0.297**	−0.242**	−0.210**	0.288**	0.260**	0.244**	0.223**
Participatory dialogue	0.312**	−0.256**	−0.260**	−0.198**	−0.204**	0.294**	0.273**	0.250**	0.226**
Behavioral confidence	0.412**	−0.261**	−0.245**	−0.220**	−0.228**	0.315**	0.297**	0.266**	0.244**
Environmental change	0.281**	−0.218**	−0.205**	−0.184**	−0.195**	0.269**	0.251**	0.235**	0.216**

** indicates *p* < 0.01.

### 3.5 Multivariate linear regression analysis of occupational exposure risk perception

Variables that were significant in the univariate analyses were further entered into a stepwise multiple linear regression model. The results showed that self-efficacy, occupational burnout, social support, occupational exposure training, regular occupational exposure drills, and supervisory oversight were significantly associated with occupational exposure risk perception (Table 3).

## 4 Discussion

### 4.1 Multilevel characteristics and theoretical implications of occupational exposure risk perception

In the field of global occupational health and patient safety research, healthcare workers’ perception of occupational exposure risk is regarded as an important factor associated with protective behaviors, institutional compliance, and the effectiveness of infection prevention and control measures (18). Risk perception is closely related to whether individuals appropriately use personal protective equipment and report exposure events in a timely manner, and may also be associated with the operational efficiency and systemic resilience of occupational safety management within healthcare institutions (19). Previous studies have primarily focused on frontline nursing staff in departments with direct patient contact, such as emergency and infectious disease units, while relatively limited attention has been paid to healthcare workers who have minimal direct patient contact but remain exposed to high-risk environments over extended periods (20).

Based on this multicenter sample, we found that occupational exposure risk perception among CSSD nurses was moderate, tending to be above the midpoint of the scale. Among the five dimensions of risk perception, behavioral confidence and perceived severity of occupational exposure consequences scored relatively high, whereas participatory dialogue scored comparatively lower. These findings suggest that CSSD nurses are generally aware of the potential hazards associated with occupational exposure and demonstrate confidence in their personal protective behaviors. However, notable deficiencies remain in risk communication, feedback mechanisms, and participatory safety management, indicating a possible disconnect between individual risk awareness and organizational-level risk communication systems.

From an interpretive perspective, the findings suggest a multilevel pattern in which individual psychological resources and organizational support factors are associated with occupational exposure risk perception. Specifically, self-efficacy and social support were positively associated with risk perception, whereas occupational burnout showed a negative association. These findings may partly reflect underlying psychological processes, including risk sensitivity, safety vigilance, and the allocation of attentional resources. Nurses with greater psychological resources may be more attuned to risk-related cues, whereas higher emotional exhaustion may be linked to lower safety vigilance, which could partly explain lower subjective risk perception (21). Occupational exposure risk perception should therefore not be viewed as related only to knowledge acquisition or technical proficiency, but rather as a comprehensive outcome associated with psychological states, work experiences, and organizational environments.

Notably, CSSD nurses are a typical occupational group within the healthcare system whose exposure risks may be underestimated or relatively overlooked. Although they have limited direct patient contact, they may still be frequently exposed to sharps injuries and residual blood or body fluids during the collection and reprocessing of contaminated instruments. A multi-department surveillance study across 36 hospitals in Shandong Province found that sharps injuries occurred mainly in clinical areas such as general wards, operating rooms, and ICUs, while the CSSD was also identified as one of the high-incidence locations, suggesting that it should not be regarded as a “low-risk setting” and should be prioritized in occupational exposure prevention and control (22). Similarly, within international surveillance systems, a Massachusetts-based sharps injury surveillance report listed “central sterile supply” as one of the departments where incidents occurred, further highlighting the practical relevance of monitoring occupational exposure in this setting (23). In addition, a survey of occupational protection practices in CSSDs across 46 hospitals in Shanghai reported an occupational exposure incidence of 35.20% (207/588), with sharps injuries being the most common type (26.36%) (24). Meanwhile, CSSD work is highly procedural and repetitive, which may to some extent attenuate the salience of risk cues, resulting in a potential mismatch between nurses’ subjective risk perception and the actual exposure context (25).

The present findings suggest that occupational exposure risk perception among CSSD nurses is associated with multiple factors, including individual psychological states, organizational support, training, and supervisory practices. Therefore, prioritizing CSSD nurses in occupational safety management and strengthening management measures around training, supervision, and organizational support may help enhance risk perception and promote more standardized protective practices. However, given the cross-sectional design of this study, these associations should not be interpreted as causal relationships, and the real-world effects of these strategies should be further evaluated through longitudinal follow-up or intervention studies.

### 4.2 Analysis of factors influencing occupational exposure risk perception among CSSD nurses

#### 4.2.1 Self-efficacy

The present study demonstrated a significant positive correlation between self-efficacy and occupational exposure risk perception among CSSD nurses, indicating that self-efficacy may be an important psychological factor associated with risk perception. Self-efficacy reflects an individual’s confidence and perceived control in completing specific tasks and represents a key psychological resource for actively coping with occupational risks. Individuals with higher self-efficacy tend to report more proactive risk recognition and protective behaviors, and this has been associated with higher levels of risk perception (26).

Previous studies have similarly confirmed the important association of self-efficacy with nurses’ occupational safety behaviors. Lan et al. (27), using structural equation modeling, found that self-efficacy was directly associated with nurses’ safety behaviors and was indirectly linked to protective compliance through risk perception. Cheng et al. (28) further reported that clinical nurses with higher self-efficacy demonstrated more proactive behaviors in sharps injury prevention, exposure reporting, and risk assessment, with significantly higher risk perception scores compared with those with lower self-efficacy. The findings of the present study are consistent with these results, suggesting that higher self-efficacy among CSSD nurses may be associated with greater sensitivity to hazard cues and more proactive protective responses; the proposed pathway linking self-efficacy, risk perception, and safety behavior warrants verification in longitudinal studies.

Evidence suggests that structured safety training is associated with higher self-efficacy and better safety-related outcomes; however, causal effects on occupational exposure risk perception should be confirmed in longitudinal or intervention studies (29). Therefore, managers may consider strengthening resources that support self-efficacy through structured training, scenario-based simulation, and peer experience sharing. Such approaches may be associated with higher risk perception and more standardized protective practices (30).

#### 4.2.2 Occupational burnout

This study found a significant negative correlation between occupational burnout and occupational exposure risk perception among CSSD nurses, indicating that higher levels of burnout are associated with lower risk perception. In high-intensity and repetitive work settings, CSSD nurses may be more likely to report emotional exhaustion and attentional blunting, which may be linked to reduced risk sensitivity and lower safety vigilance (31). Previous research has also suggested that occupational burnout may be associated with poorer safety behaviors through lower work engagement and cognitive alertness (32). Quesada et al. (33) reported a significant negative association between burnout and risk perception among nurses, suggesting that emotional exhaustion may be associated with a reduced ability to recognize and respond to warning signals. Xie et al. (34) further indicated that burnout may also be indirectly associated with lower frequencies of safety behaviors through reduced self-efficacy and protective motivation, findings that align with the present study. Collectively, these results suggest that occupational burnout may be associated with lower nurses’ risk appraisal capacity and willingness to engage in protective behaviors, which may be linked to lower levels of risk perception.

Therefore, greater attention should be paid to the psychological health and work-related support of CSSD nurses. Measures such as job rotation with adequate rest, psychological counseling, team communication, and humanistic care may help alleviate emotional exhaustion. In addition, performance appraisal systems could incorporate the principle of giving equal weight to psychological well-being and safety behaviors, potentially helping mitigate burnout and providing a basis for future longitudinal evaluations of how changes in burnout may be related to changes in risk perception over time.

#### 4.2.3 Social support

The present study revealed a positive correlation between social support and occupational exposure risk perception among CSSD nurses. Individuals with higher social support tend to report higher levels of risk perception and may have better access to information, emotional regulation, and coping resources, which may in turn be associated with more standardized protective practices. Social support encompasses three dimensions—subjective support, objective support, and support utilization—and is closely related to safety awareness and confidence in protective behaviors at both psychological and behavioral levels. Adequate organizational and interpersonal support may buffer occupational stress to some extent and may be associated with a greater ability among nurses to perceive and respond to hazardous events (35). Gillet et al. (36) reported that higher levels of social support within nursing units were associated with greater job satisfaction and better safety performance. Similarly, Al Ma’mari et al. (37) found that positive perceptions of organizational support and peer collaboration were associated with higher nurses’ awareness of safety culture and occupational risks, whereas insufficient support and communication barriers were associated with lower risk vigilance and more unsafe behaviors. These findings are consistent with the results of the present study.

Accordingly, management could prioritize safety culture development by strengthening leadership engagement, peer support, and professional support networks. Practical approaches may include implementing an occupational safety mentorship scheme, establishing clear and accessible channels for risk reporting and feedback, and organizing regular peer exchanges on protective practices. These initiatives may enhance nurses’ sense of belonging and perceived support and may be associated with more proactive occupational exposure risk perception and related protective behaviors. From an SEM perspective, social support is not only an individual-level psychological resource but also, to some extent, may reflect organizational safety culture and managerial support. Higher organizational support and stronger peer interactions may be linked to greater attention to occupational risks and higher safety vigilance, suggesting that risk perception may extend beyond individual cognition to a shared team norm. A supportive environment may also be associated with more sustained adherence to standardized protective practices.

#### 4.2.4 Occupational exposure training

Our findings showed that receiving occupational exposure–related training was associated with higher levels of risk perception. Consistently, previous research has reported that training experience is also correlated with indicators such as greater protective knowledge and better awareness of exposure reporting and management procedures (38). Previous studies have shown that nurses who receive high-quality occupational protection training tend to demonstrate greater risk awareness and behavioral compliance in the prevention of sharps injuries, chemical exposure, and biological contamination, with significantly higher exposure reporting rates and adherence to protective protocols compared with those who have not received systematic training (39). The association of training with risk perception may extend beyond knowledge acquisition; by strengthening self-efficacy and reinforcing a team-based safety culture, training may also be associated with protective practices, although these relationships should be tested in longitudinal or intervention studies.

Accordingly, CSSD managers may consider establishing a standardized, continuous, and context-specific occupational exposure training system, incorporating protective knowledge and operational protocols into onboarding training, annual refresher training, and mandatory modules prior to the implementation of new techniques (40). Training may also be complemented by debriefing of typical incidents and case-based learning to enhance its relevance and practicality. In addition, digital platforms can be leveraged to deliver online learning and competency assessments, enabling dynamic updates of safety knowledge and traceable management of training records (41). Moreover, linking training to performance appraisal and reward-penalty mechanisms may be associated with greater engagement and clearer accountability for protective practices, which may contribute to a safety culture in which protection is prioritized and risks are proactively identified; the potential impact on risk perception and protective behaviors warrants evaluation (42).

#### 4.2.5 Regular occupational exposure drills

Our results showed that nurses who regularly participated in occupational exposure emergency drills had higher risk perception scores than those who did not, indicating a positive association between drill participation and risk perception. Participation in drills may be associated with higher situational awareness and preparedness (e.g., better exposure recognition and familiarity with response procedures), which may co-occur with more standardized protective practices. As an extension of occupational protection training, drills can be used to simulate real exposure scenarios and may provide opportunities to practice risk identification, judgment, and emergency response skills under pressure; whether drills are associated with sustained behavioral change should be evaluated in prospective or intervention studies. Compared with traditional didactic training, scenario-based drills may be more likely to be associated with psychological alertness and crisis response awareness, potentially contributing to a shift in risk perception from “passive cognition” to “active defense,” while also being associated with greater protective confidence and team collaboration (43).

Evidence suggests that scenario-based drills may be associated with better judgment and response capacity under pressure and may also be linked to more proactive prevention, although this requires confirmation in prospective studies. Such drills have also been associated with greater protective knowledge, stronger risk vigilance, and better adherence to operational standards among nurses (44). Accordingly, CSSD managers may consider instituting regular drill programs and delivering scenario-based training informed by typical cases and retrospective reviews of risk events to support nurses’ practical response capacity and team coordination. Such strategies may be associated with greater attention to occupational risks and higher safety vigilance, and may be associated with higher and more stable levels of risk perception.

#### 4.2.6 Supervisory oversight of protection

The results of this study indicate that CSSD nurses who received supervisory oversight of protective behaviors had significantly higher levels of occupational exposure risk perception than those without such oversight. Multivariate regression analysis further showed that supervisory oversight was positively associated with risk perception. Through behavioral modeling, feedback, and reinforcement of institutional norms, supervisory oversight may serve as an organizational cue and may be associated with greater attention to occupational hazards and stronger protective awareness. This association may reflect heightened cognitive vigilance toward risk and more consistent protective behaviors in daily practice (45). Previous research (46) reported that leadership behaviors characterized by continuous communication, positive feedback, and role modeling were associated with greater acceptance of safety management and higher perceived risk awareness and protective consciousness. Leadership may also be associated with a positive safety climate and with more proactive risk identification and response during routine work, which has been linked to better safety-related outcomes in prior research. Consequently, in high-risk operational settings such as CSSDs, managers may consider strengthening on-site supervision and feedback, integrating risk oversight into routine management processes, and establishing a closed-loop mechanism of “supervision–feedback–improvement”; the potential effects of these strategies on risk perception warrant evaluation in future studies.

Taken together, organizational-level factors, such as occupational exposure training, emergency drills, and supervisory monitoring, were associated with risk perception. These findings suggest that a more supportive organizational climate and stronger institutional arrangements may contribute to greater risk awareness and better occupational protection awareness among CSSD nurses. From a practice perspective, these strategies warrant further strengthening and refinement. Future longitudinal and intervention studies are needed to better delineate their potential effects on risk perception and protective behaviors.

In summary, guided by the SEM framework, this study systematically examined factors associated with occupational exposure risk perception among CSSD nurses. The findings suggest that risk perception is unlikely to be explained by a single factor; rather, it may reflect a combined pattern of influences across multiple levels, including individual psychological resources, interpersonal support, and organizational management. At the individual level, self-efficacy was positively associated with risk perception, whereas occupational burnout was negatively associated with risk perception; the underlying psychological mechanisms (e.g., safety vigilance and risk appraisal tendencies) warrant further investigation. At the interpersonal and organizational levels, receiving occupational exposure training, participating in emergency drills, and supervisory monitoring were statistically associated with risk perception, indicating that supportive management and practice environments may accompany greater risk awareness and protective consciousness. Although some policy-level variables were not statistically significant in the multivariable mode, their significance in univariate analyses suggests that the broader institutional context may be related to inter-individual differences in risk perception and may be indirectly associated with risk perception through organizational management mechanisms and behavioral norms. Overall, these findings provide preliminary evidence for understanding the multilevel correlational structure of occupational exposure risk perception among CSSD nurses and offer directions for future longitudinal or intervention studies focusing on psychological resource support, optimization of organizational support, and institutional development.

## 5 Limitations

This study still has several limitations. First, this study used a cross-sectional design, and all variables were measured at the same time point; therefore, the findings only reflect associations between variables and do not allow inference regarding causal direction or temporal pathways. Second, data were collected through an online self-administered questionnaire. Although participation was voluntary and anonymous, and the survey link was distributed uniformly by hospital liaisons without individual-level screening, selection bias, as well as social desirability bias and recall bias, cannot be completely ruled out. Third, the sample was drawn from 28 tertiary hospitals in Chongqing, and participating hospitals were recruited using a feasibility-based convenience approach rather than probability sampling. Although this strategy facilitated the implementation of the multicenter survey and allowed the inclusion of hospitals with varying institutional characteristics, it may also have introduced selection bias, thereby limiting the representativeness of the sample and affecting the external validity of the findings. Therefore, caution is warranted when generalizing these results to CSSD nurses in other regions, at different hospital levels, or in other healthcare settings. Future studies are needed to further validate these findings using probability-based or stratified sampling across broader geographic areas and more diverse types of healthcare institutions, in order to improve sample representativeness and the generalizability of the conclusions. Fourth, variables at the interpersonal, organizational, community, and policy levels were measured using contextualized items developed for this study, most of which were dichotomous. In addition, validated safety culture instruments and qualitative data were not incorporated. As a result, the complexity of organizational and contextual influences may not have been fully captured, which may have contributed to an underestimation of organizational-level effects and may have limited a more in-depth interpretation of the underlying mechanisms. Future studies should adopt probability sampling or stratified sampling across broader regions and more diverse healthcare settings, integrate objective indicators and mixed-methods designs, and employ more refined multi-item scales to further enhance representativeness, measurement validity, and explanatory depth.

## 6 Conclusion

Guided by the SEM framework, this multicenter survey included 580 CSSD nurses from 28 tertiary hospitals in Chongqing. Overall, occupational exposure risk perception was moderate, tending to be above the midpoint of the scale, but there remains room for improvement. Further analyses identified multilevel correlates. At the individual level, self-efficacy and social support were positively associated with risk perception, whereas occupational burnout was negatively associated. At the organizational/managerial level, receiving occupational exposure training, regular occupational exposure drills, and supervisory monitoring of protective practices were associated with higher risk perception.

Based on these findings, a coordinated multilevel approach may be considered, and its effects should be evaluated. At the individual level, strengthening psychological resource support and implementing stress-reduction strategies may help enhance self-efficacy and alleviate burnout. At the organizational level, reinforcing structured training and scenario-based drills, strengthening supervision and feedback mechanisms, and continuously optimizing occupational protection systems and safety culture may facilitate sustained improvements in both risk perception and protective behaviors. Future multicenter longitudinal follow-up or intervention studies are warranted to further clarify causal relationships and to evaluate the effectiveness of multilevel interventions.

## Data Availability

The datasets presented in this article are not readily available because of ethical and privacy restrictions. Requests to access the datasets should be directed to 1053085055@qq.com.
